# A clinical protocol for the effect of acupuncture combined with qianliean suppository on inflammatory factors in patients with chronic prostatitis

**DOI:** 10.1097/MD.0000000000027913

**Published:** 2021-12-23

**Authors:** Yi Lei, Hong-hong Du

**Affiliations:** a The Second Affiliated Hospital of Shaanxi University of Chinese Medicine, Xianyang, Shaanxi, P. R. China; b The Affiliated Hospital of Shaanxi University of Chinese Medicine, Xianyang, Shaanxi, P. R. China.

**Keywords:** acupuncture, chronic prostatitis, protocol, randomized controlled trial

## Abstract

**Introduction:**

Chronic prostatitis (CP) is a common genitourinary disorder in adult men. It has a high incidence, a complex disease, and a lingering course, which seriously affects the quality of life of patients. For the treatment of CP, the currently available treatment methods are limited and patients are not satisfied with the treatment results. Therefore, more effective treatment options need to be further explored.

**Methods:**

The study is a single-blind, parallel-group, randomized controlled clinical trial consisting of a 4-to-6-week treatment period and a 6-month follow-up period. Included participants will be randomized into three groups and given a treatment regimen of acupuncture, qianliean suppository, respectively. Patients in each group will be treated for 1 month as a course of treatment. The clinical efficacy and changes in inflammatory factor levels in each group will be assessed at the end of treatment.

**Discussion:**

The trial aims to promote a more effective, standardized, and efficacious treatment protocol for CP in the clinical setting.

## 1 Introduction

Chronic prostatitis (CP) is the most common genitourinary disorder in adult men. Surveys have shown that 35% to 50% of men will be affected by prostatitis at some point in their lives.^[1]^ CP is a difficult class of disease to treat. The typical clinical presentation of the disease is prolonged and recurrent pain in the pelvic region. The duration of the painful discomfort is often greater than 3 months. Some patients also often have varying degrees of urinary symptoms and sexual dysfunction.^[2,3]^ CP is a complex and protracted disease, which is difficult to treat. It brings serious psychological and economic burdens to patients and seriously affects their quality of life. Some patients even develop severe anxiety and depression as a result, which in turn leads to many social problems. Currently, it has attracted the attention of clinicians in various countries. In recent years, Western medical treatment of CP has gradually changed from antibiotics to a combination of α-blockers, androgens, anti-anxiety and antidepressants, and the efficacy has improved, but patients are still not satisfied with the treatment effect.^[4]^ Therefore, it is often necessary to continuously increase the dose of medication in order to achieve a more satisfactory therapeutic effect. This may cause serious adverse effects and affect patient compliance with treatment. Based on the above status, more effective treatment modalities need to be further explored.^[5]^ As a traditional treatment modality inherited for thousands of years, acupuncture has been valued and widely used in the clinical treatment of CP in recent years.^[6]^ This treatment modality can improve the quality of life by promoting neuromuscular excitability and by enhancing the contractility of urethral muscles and perineal muscles. qianliean suppository is a topical preparation made from several Chinese herbs with the effects of clearing heat, relieving dampness, promoting laxity, resolving siltation, dispersing nodules, and relieving pain. This study will investigate the therapeutic effects of acupuncture combined with qianliean suppository on CP, aiming to provide valuable evidence-based medical evidence for clinicians.

## 2 Methods

### 2.1 Study design and setting

**Figure 1:**
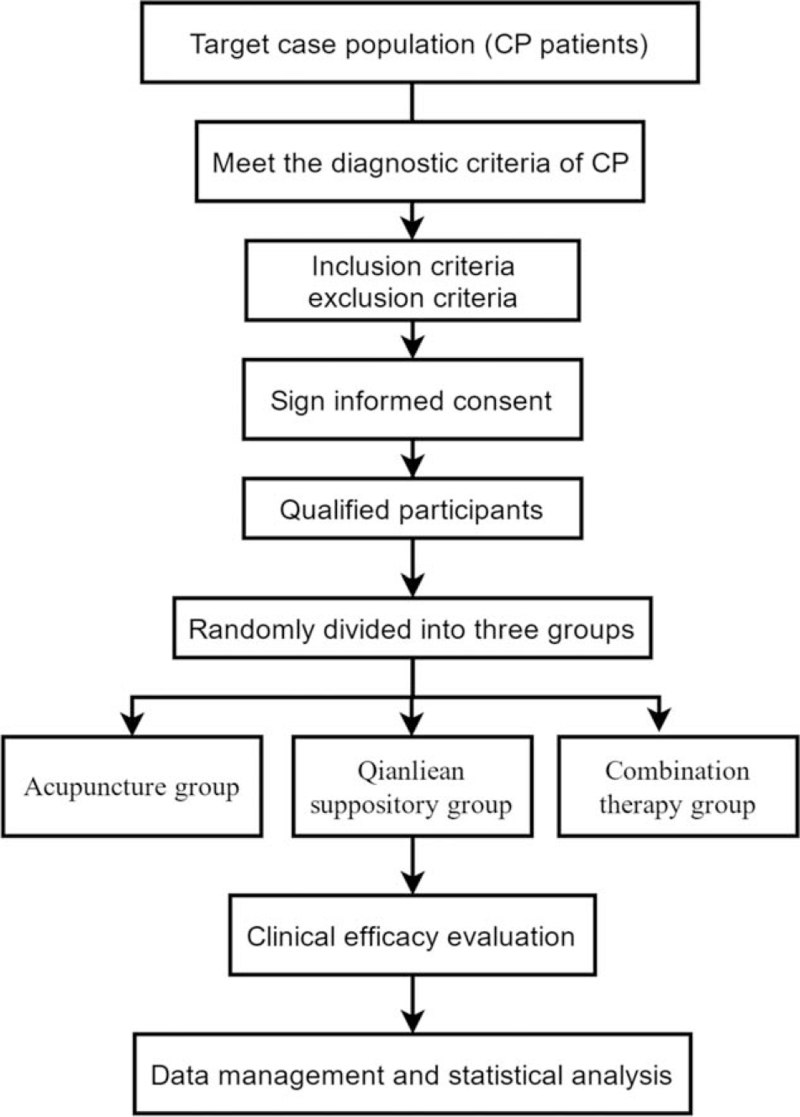
Study design flow chart.

The study is a single-blind, parallel-group, randomized controlled clinical trial consisting of a 4- to 6-week intervention and a 6-month follow-up period. The study will be conducted at one site, the Affiliated Hospital of Shaanxi University of Chinese Medicine. A brief flow chart of the entire study is shown in Figure 1. This study will adopt a parallel controlled design approach. We will ensure a balance of baseline data across groups by having an adequate sample size and a completely randomized grouping method. The study will be approved by the Ethics Committee of the Affiliated Hospital of Shaanxi University of Chinese Medicine. We will not begin recruiting participants until ethics committee approval has been obtained.

### 2.2 Participants

#### 2.2.1 Diagnostic criteria of CP

The diagnostic criteria for this study will be developed with reference to s *Wu Jieping Urology (2013 edition)*.^[7]^ The specific criteria are as follows:

(1) Symptoms: They are divided into two aspects. On the one hand is the symptom of irritation in the lower urinary tract, and the other is the symptom of inflammatory reaction or reflex pain. The symptoms are varying degrees of urinary frequency, urgency, painful urination, incomplete urination, and burning in the urethra. There is occasionally a small amount of white discharge from the urethra in the morning, at the end of urination or during bowel movements. Pain and swelling in the perineum, external genital area, lower abdomen, suprapubic area, lumbosacral and perianal areas.
(2) Prostate palpation: texture: full gland, or soft and hard, or inflammatory nodules, or tough texture. Pressure pain: there may be limited pressure pain. Size: can be enlarged, normal or reduced.
(3) Prostate fluid (EPS) Microscopy: WBC 10/HP; lecithin vesicles reduced or absent.
(4) Bacterial culture of prostatic fluid (EPS): negative bacterial culture.
(5) Ultrasonography: Ultrasound image is obviously abnormal: manifesting as normal or reduced prostate size, uneven internal echogenicity, visible enhanced light spots and nodal echogenicity, enhanced, thickened and rough peritoneal echogenicity. The ultrasound image is mildly abnormal; it shows normal or slightly enlarged prostate size, slightly strong or weak internal echogenicity, and poorly defined periosteal echogenicity.

The diagnosis of CP can be made by having (1) and (2) above, and other tests can be performed according to the patient's specific situation.

#### 2.2.2 Eligibility criteria

The inclusion criteria for participation are as follows:(1) Patients who mEet the diagnostic criteria for CP; (2) Patients who have a medical history of at least 3 months and are not on the appropriate treatment medication within 2 weeks prior to admission; (3) Patients who voluntarily participate in this study and signe an informed consent form. (4) Patients should be between the ages of 18 and 50 years; (5) Patients can receive emails and cell phones can receive text messages.

The exclusion criteria are as follows:(1) patients with combined severe neurological dysfunction, urethral stricture, prostate tumors; (2) patients with a history of pelvic surgery; (3) patients with combined severe cardiovascular disease, cerebrovascular disease, hepatic and renal insufficiency, hematopoietic system disease, and neurological disease; (4) patients with allergic reactions to drugs and psychiatric disorders.

#### 2.2.3 Recruitment

The study will be open to participants from all regions of China. Participants can enroll through the hospital's official website or be referred to our specialty clinics by healthcare professionals. The study will be promoted to healthcare providers, patient organizations, and directly to the public through the website and social media. We will also advertise in digital media. Participants will be assigned a screening ID and a member of the study team will conduct the initial eligibility screening. If they are interested in participating and potentially eligible, participants will be booked for the assessment. The inclusion assessment will be conducted under the supervision of a clinician. Additional information about the study will be provided immediately following this assessment. The assessor will perform final verification of the participant's eligibility criteria and sign the informed consent form. If the assessor is unsure of the eligibility of a potential participant, the principal investigator will have the final say. After signing the informed consent form, participants will be assigned a new study ID and will be randomized to a group. Treatment will begin within 1 week of randomization grouping. Where possible, excluded participants who still require clinical attention will be given routine treatment. Screening IDs and reasons for exclusion will be recorded for use in the final study report.

### 2.3 Randomization, allocation concealment, and blinding

The randomization system used for this study will be provided by the Affiliated Hospital of Shaanxi University of Chinese Medicine. Eligible patients will be identified by the treating physician based on inclusion and exclusion criteria. Participants will be referred to a study coordinator who will randomly assign them to each group. The randomization list will be kept by the statistician and study coordinator until the end of the study to ensure allocation concealment.

### 2.4 Interventions

(1) Acupuncture group: selected acupoints: *Shensu, Zhongliao, Huiyang, and Sanyinjiao* (all taken bilaterally). The acupuncture points will be positioned with reference to the 2006 National Standard of the People's Republic of China (GB/T 12346–2006) “Name and Positioning of Acupuncture Points”. After local sterilization of the acupoints, Shensu and Sanyinjiao will be needled with 0.3 × 40 mm milli-needles at a depth of 20 to 30 mm; Zhongliao and Huiyang will be needled with 0.3 × 75 mm milli-needles at a depth of 50 to 70 mm; the needles will be retained for 30 minutes.
(2) Qianliean suppository group: use prostate suppository (National Drug Administration Z10980066, specification 2g × 5 s) for treatment. This product is brown or black-brown torpedo type suppository. Usage: The drug is inserted into the anus at night before bedtime, placed at a depth of 2 cm, 1 capsule each time, once a day. The ingredients of this drug are: extracts of Chinese herbs such as *Huangbai, Huzhang, Zhizi, Dahuang, Zelan, Maodongqing, Wuzhuyu, Weilingxian, Shichangpu and Lizihe*. Precautions for use: After the suppository is inserted into the anus, if there are uncomfortable symptoms such as bowel movement, abdominal pain and diarrhea, the method of use can be improved, such as applying vegetable oil outside the suppository or placing the suppository deeper, after the rectum adapts, the above symptoms can be reduced or disappear.
(3) Combination therapy group: The treatment methods of (1) and (2) are combined.

Patients in each of the above groups will be treated for 1 month as a course of treatment. Patients in each group will be asked to abstain from sexual intercourse during the treatment period, to ensure the cleanliness of the perineal area, to observe the adverse reactions and to provide timely and effective treatment to ensure the treatment effect.

### 2.5 Outcome measures

#### 2.5.1 Primary outcome measure

The CP symptom index (NIH-CPSI) with prostatitis-related sexual function index score will be performed before and after treatment. The changes of urodynamic indexes will be observed in each group before and after treatment, and the specific indexes included maximum urinary flow rate, maximum bladder capacity, maximum forced urinary muscle pressure and bladder stability. The observation times for the primary outcome indicators are shown in Table 1.

**Table 1:** Treatment schedule and outcome measures.

Items	Before Treatment	Treatment period, twice a week, 8 times in total (treatment starts within 1 wk after registration)				Post observation Period
TIME POINT	Registration	wk 1	wk 2	wk 3	wk 4	1 wk later Treatment completion
Inclusion criteria	√					
Exclusion criteria	√					
Informed consent	√	√	√	√	√	√
Confirmation of subjective symptoms	√		√		√	√
Chronic prostatitis symptom index	√	√	√	√	√	√
prostatitis-related sexual function index Observation of adverse events	√		√		√	

Clinical efficacy evaluation criteria: significant effect: the symptoms of urinary disorder disappeared after treatment, no pain and discomfort, and normal sexual function will be restored at the same time; effective: the symptoms of urinary disorder basically disappeared, a little pain and discomfort existed, pain will be tolerated, and sexual function will be basically restored to normal; ineffective: the situation of urinary disorder did not improve, obvious pain and discomfort existed, and no significant change in sexual function will be seen. The total effective rate =  (the number of effective cases to the number of effective cases)/total cases × 100%.

#### 2.5.2 Secondary outcome measures

Four ml of fasting elbow venous blood will be collected from the participants before and after treatment, and the blood will be shaken in a tube with anticoagulant, centrifuged at 3000 r/min for 10 min and tested immediately. Serum inflammatory factors, including interleukin (IL)-1β, IL-6 and tumor necrosis factor-a (TNF-a), will be measured by enzyme-linked immunosorbent assay in each group of patients.

### 2.6 Date collection and management

All enrolled participants must complete a case observation form. All items on the observation form must be recorded carefully and in detail for all patients who have been screened and qualified to enter this trial and have completed the informed consent form. Full-text Analysis Set: For all cases that are randomized and grouped and received at least one treatment, the data of those cases in which the full treatment course is not observed are carried forward to the final results of the trial using the last observation data for intentional analysis of the main efficacy indicators. Per-Protocol Set: All cases that conformed to the trial protocol, had good compliance, did not take prohibited drugs during the trial, and completed the case report form, are statistically analyzed for all efficacy indicators. Safety Set: All cases that were randomized, completed at least one treatment, and had post-treatment safety evaluation data constituted the safety analysis population for this study.

### 2.7 Statistical analyses

The case information collected by Excel will be imported into SPSS 25.0 statistical software to establish the analysis database and complete the statistical analysis of the data. Normality test will be performed by Kolmogorov-Smirnov method; chi-square test will be performed by Levene method. The measurement data conform to normal distribution and will be described by means and standard deviations, and comparisons between groups will be made by one-way ANOVA ANOVA or independent samples *t*-test; two-way comparisons will be made by Bonferroni method corrected *P*-value; not conforming to normal distribution, the measurement data will be described by medians and quartiles, and comparisons between groups will be made by Kruskal-Wallis method test or The Mann-Whitney *U* test will be used for comparison between groups, and the Dunnett's method will be used for comparison between two groups. The dichotomous data will be expressed as composition ratio and frequency in describing the distribution pattern, and the chi-square test and exact Fisher test will be used for comparison between groups. NIH-CPSI scores, mean urine flow rate and maximum urine flow rate will be compared between the two groups of patients at different time points using repeated measures ANOVA-generalized linear model equations.

## 3 Discussion

The exact cause of CP is not known. However, in some patients, symptoms may remain unchanged over time, and in others they may worsen further. Epidemiological studies have shown that the high incidence of CP is in the age groups of 30 to 40 and 61 to 70 years. The incidence of the disease increases yearly in high-incidence settings.^[8]^ CP mostly presents with prolonged and recurrent pain or discomfort in the pelvic region, often lasting more than 3 months. Some patients also suffer from varying degrees of urinary difficulties and sexual dysfunction, which seriously affects the quality of life of patients.^[3]^ Therefore, effective treatment is needed. However, the use of medication alone does not improve the patient's symptoms significantly, and it is difficult to achieve the standard of cure. And long-term medication is also often prone to a series of adverse reactions. Therefore, the adoption of more effective methods for treating the disease is also an important topic of extensive clinical research. Current studies have shown that the occurrence of CP is strongly associated with inflammatory factors.^[9]^ IL-1β, IL-6, and TNF-a show high expression in CP. IL-1β is an important component of the IL-1 receptor, and its level increases significantly when the body is stimulated by a stress response; IL-6 is a lymphokine produced by activated T lymphocytes and fibroblasts, which promotes the growth and differentiation of primary bone marrow-derived cells and enhances natural killer cell lysis capacity. TNF-α is a cytokine that can directly kill tumor cells without significant toxicity to normal cells. TNF-α appears earliest after the inflammatory response, mainly increases vascular endothelial cell permeability, regulates other tissue metabolic activities, and promotes cytokine synthesis and release.^[10]^

For the treatment of this disease, the common treatment options can be divided into pharmacotherapy (α-blockers, antibiotics, analgesics and botanicals), physiotherapy (biofeedback, microwave therapy, acupuncture) and surgery. Several randomized controlled trials and Meta-analyses have shown acupuncture to be effective in CP^[11–13]^ and a relatively safe treatment. Studies have shown that acupuncture can improve patients’ symptoms, reduce patients’ NIH-CPSI scores and decrease the number of white blood cells.^[14]^ However, there is still a lack of systematic and comprehensive clinical studies on acupuncture for the treatment of this disease, and most trials lack follow-up to understand the long-term efficacy. Therefore, this study will objectively evaluate the short-term and long-term clinical efficacy of different treatment regimens and the effect on the level of inflammatory factors in the organism by designing a standardized clinical trial with an acupuncture group and a Qianliean suppository group with NIH-CPSI scale score as the main outcome index, combined with a long follow-up. This study hopes to provide more ideas for the clinical treatment of CP.

## Author contributions

**Data curation:** Yi Lei.

**Funding acquisition:** Hong-hong Du.

**Project administration:** Hong-hong Du.

**Resources:** Yi Lei, Hong-hong Du.

**Software:** Yi Lei.

**Supervision:** Hong-hong Du.

**Writing – original draft:** Yi Lei.

**Writing – review & editing:** Hong-hong Du.
